# Dr. Parry, on the Circulation of the Blood

**Published:** 1816-11

**Authors:** C. H. Parry


### Medico-Chirurgical
### Journal and Review.

VOL. II.] NOVEMBER, 1816. [NO. 11.

### PART I.

### ORIGINAL COMMUNICATIONS.

——— quid utile ———

*To the Editors of the Medico-Chirurgical Journal & Review.*

GENTLEMEN, Bath, Sept. 10, 1816.

IT appears to me, that your first objection to my opinion of the want of dilatation and contraction of the larger arteries, during the systole and diastole of the left ventricle of the heart, arises from a supposition, that the arterial system contains more blood during that systole than during the diastole. This supposition, if you will consider the structure of the whole sanguiferous circle, you will, I think, readily see to be unfounded.

In order to facilitate the discussion of this question, we will, if you please, put the smaller circulation, or that through the lungs, completely out of the question, and consider the heart as only bilocular, consisting of one auricle and one ventricle. We will also suppose, what is sufficiently near the truth, for our present inquiry, that the capacity of the auricle and ventricle is equal, and that the quantity of blood in the whole circulating system remains the same.

Since, therefore, the contractions of the auricle and ventricle are precisely alternate, and each contraction of the latter is precisely synchronous with each dilatation of the former, it is obvious that, during the systole of the ventricle, the same quantity of blood will be in the vessels and auricle, as in the vessels and ventricle during the diastole of the ventricle.

Hence, by the foregoing datum respecting the relative size of the auricle and ventricle, the quantity of blood in the vessels will always be precisely the same; and no necessity whatever for the dilatation of the arteries is produced by the systole of the heart.

That, at each systole of the ventricle, the blood is impelled with great force against the sides of the arteries, is readily admitted. I have, however, shewn, that those vessels, during their natural state, are always stretched beyond that degree, which they would spontaneously assume by their conjoint contractile powers; and the question is, whether the impulse given by the usual systole of the ventricle be sufficient to increase that distention. The evidence of eye-sight, assisted by very strong magnifying powers, has, in numerous instances, convinced me, and many other persons with me, that it is not. I confess myself greatly inclined, on this occasion, to adhere to the old maxim, "De non apparentibus et non existentibus eadem est ratio;" and it is surely a rather dangerous mode of reasoning, to infer an effect from the operation of any cause which is itself doubtful, when the existence or non-existence of that effect can be brought to the unequivocal test of ocular evidence.

With regard to the velocity of the impulse, of which you speak, we know it to be very little, comparatively with what the eye is perfectly capable of accurately perceiving. According to the largest estimate, the blood is not thrown out of the human left ventricle into the aorta, at a greater rate than 150 feet in a minute, or 2½ feet in a second; not $\frac{1}{6}$ of the velocity with which a flea makes a leap, which a good eye will often follow. Besides which, what difficulty is there in seeing the dilatation, which the systole of the ventricle causes actually to exist, whenever an artery is, to a certain degree, contracted by any compressing substance?

With regard to the successive propagation of impulse along the several columns of blood, I believe with Bichât, that this is virtually chimerical. I consider the blood-globules, buoyant in the fluid in which they swim, as, for most purposes of reasoning, incompressible; and, in this view, they are like a series of billiard-balls in close contact with each other, so that, if an impulse is made from behind, immediately the foremost ball, however distant, is moved; or, being already in motion, as in the present instance, feels the preponderating velocity of the new impulse, to all sensible purposes synchronously with all those which are beyond it.

With respect to the continuance of the passage of a fluid through a passive tube, the upper end of which is hermetically closed, after the first mover has ceased to act, I see no reason whatever to doubt the possibility of that fact. I know, indeed, that if a tube of a certain small diameter be filled with water, and the thumb be placed on the top of that tube, so that there shall be no air between the thumb and the water, the water will not run out of the open end of that tube, though it be placed downwards. This fact is easily enough explained; but I cannot see how it applies to the case before us, which is that of a full tube, forming a circle closed at both ends, and in which, it seems to me, that the fluid must continue to move for a certain time after the power, which has impelled it, has ceased to act, just as an arrow continues to fly after it has left the bow; and, indeed, must fly, until the resistance shall have become equal to the impulse. In the example with which we are concerned, the time required for the continuance of this movement is very short; for the impulse by the systole is usually renewed oftener than once in a second; and it is the increase of momentum by that very renewal, which we so plainly perceive under the form of what we call the pulse.

That I do not believe any part of the arterial system to be empty during healthy circulation, you very justly conclude; and yet you doubt how the case can be otherwise, suppose in the beginning of the aorta, unless that artery contracts upon the smaller quantity of blood which remains in it during the diastole of the ventricle.

In this objection it appears to me that you admit of two hypotheses; first, that the vessel is expanded by the systole; and, secondly, as before stated, that there is in it more blood during the systole than during the diastole.

With regard to the expansion of the root of the aorta, I have already admitted, in a former work, that I have nothing to say in point of fact. It should seem that the muscular fibres, which thinly supply that part, are intended as a provision against somewhat of that kind, which may, at least casually, occur. If, however, this be admitted, it is no argument for a similar change in other parts of the arterial system.

But neither an expansion and contraction even of the root of the aorta, nor consequently an alternate increase and diminution of the quantity of blood in that or any other artery, are at all necessary for the removal of your difficulty. When the left ventricle contracts, it expels its blood, as for example two ounces, into the aorta; but it does not, as I have before explained, add that quantity to what existed before. It only displaces an equal quantity, causing the whole mass to advance towards the right auricle; which, at the same instant, receives just as much of the mass as was supplied by the ventricle. In this case, what need is there of either fulness or dilatation in the first moment, or systole; or of emptiness or contraction in the second moment, or diastole of the ventricle?

With regard to the semilunar valves of the aorta, they shut on the diastole, in consequence of the resistance — not a resistance produced by any contraction of the arterial coats, but that which is opposed by the column of blood beyond them, which moves with a less velocity, in proportion as the preponderance of impulse from the action of the ventricle has diminished.

It is, however, I believe, now generally admitted by physiologists, that these valves of the aorta do not, during the expansion of the ventricle, wholly prevent some regurgitation of blood into its cavity; nor, indeed, does such a degree of accuracy seem to be à priori necessary.

With regard to any indefinitely minute degree of dilatation of arteries, which the mind might conceive to arise from each systole of the ventricle, but which the senses cannot detect, it will readily be acknowledged that my work has no bearing whatever on such a view of the subject. I was there considering merely the sensible share which such a cause had in producing the phænomenon of the pulse.

C. H. PARRY.

